# Ten-year patient-reported outcomes following total and minimally invasive unicompartmental knee arthroplasty: a propensity score-matched cohort analysis

**DOI:** 10.1007/s00167-016-4404-7

**Published:** 2016-12-29

**Authors:** Edward Burn, Maria T. Sanchez-Santos, Hemant G. Pandit, Thomas W. Hamilton, Alexander D. Liddle, David W. Murray, Rafael Pinedo-Villanueva

**Affiliations:** 1 Nuffield Department of Orthopaedics, Rheumatology and Musculoskeletal Sciences, Botnar Research Centre, University of Oxford, Windmill Road, Oxford, OX3 7LD UK; 2 Arthritis Research UK Centre for Sport, Exercise and Osteoarthritis, University of Oxford, Oxford, UK; 3 Nuffield Orthopaedic Centre, Oxford University Hospitals NHS Foundation Trust, Windmill Road, Oxford, OX3 7LD UK; 4 Institute of Orthopaedics and Musculoskeletal Sciences, Royal National Orthopaedic Hospital, University College London, Stanmore, Middlesex HA7 4LP UK; 5 MRC Lifecourse Epidemiology Unit, Southampton General Hospital, University of Southampton, Tremona Road, Southampton, SO16 6YD UK

**Keywords:** Arthroplasty, Knee replacement, Knee prosthesis, Knee osteoarthritis

## Abstract

**Purpose:**

For patients with medial compartment arthritis who have failed non-operative treatment, either a total knee arthroplasty (TKA) or a unicompartmental knee arthroplasty (UKA) can be undertaken. This analysis considers how the choice between UKA and TKA affects long-term patient-reported outcome measures (PROMs).

**Methods:**

The Knee Arthroplasty Trial (KAT) and a cohort of patients who received a minimally invasive UKA provided data. Propensity score matching was used to identify comparable patients. Oxford Knee Score (OKS), its pain and function components, and the EuroQol 5 Domain (EQ-5D) index, estimated on the basis of OKS responses, were then compared over 10 years following surgery. Mixed-effects regressions for repeated measures were used to estimate the effect of patient characteristics and type of surgery on PROMs.

**Results:**

Five-hundred and ninety UKAs were matched to the same number of TKAs. Receiving UKA rather than TKA was found to be associated with better scores for OKS, including both its pain and function components, and EQ-5D, with the differences expected to grow over time. UKA was also associated with an increased likelihood of patients achieving a successful outcome, with an increased chance of attaining minimally clinically important improvements in both OKS and EQ-5D, and an ‘excellent’ OKS. In addition, for both procedures, patients aged between 60 and 70 and better pre-operative scores were associated with better post-operative outcomes.

**Conclusion:**

Minimally invasive UKAs performed on patients with the appropriate indications led to better patient-reported pain and function scores than TKAs performed on comparable patients. UKA can lead to better long-term quality of life than TKA and this should be considered alongside risk of revision when choosing between the procedures.

**Level of evidence:**

II.

**Electronic supplementary material:**

The online version of this article (doi:10.1007/s00167-016-4404-7) contains supplementary material, which is available to authorized users.

## Introduction

For individuals with end-stage arthritis of the knee, joint replacement relieves pain and improves function [8]. In patients with anteromedial arthritis, either a total knee arthroplasty (TKA) or a unicompartmental knee arthroplasty (UKA) can be undertaken, where the entire joint or only the affected compartment are replaced, respectively [1, 33].

Patient-reported outcome measures (PROMs) capture patients’ perceptions of their own functional status and well-being. PROMs can focus on patients’ perceptions of their general health or of health related to specific diseases or conditions [10]. While condition-specific measures may be more sensitive, generic measures are required for informing resource allocation decisions across a health system [15, 25]. Since 2009, PROMs have been routinely collected for all patients receiving a knee arthroplasty funded by the National Health Service (NHS) in England [12], with the condition-specific Oxford Knee Score (OKS) and the generic EuroQol 5 Domain (EQ-5D) recorded pre-operatively and 6 months post-operatively. After six months, UKA has been found to result in better OKS and EQ-5D, with those receiving UKA more likely to achieve the very best outcomes [29]. Whether these improvements are maintained in the longer-term, however, is unknown.

The choice between UKA and TKA is likely to also have long-term implications for patient-reported health outcomes. Whether the observed differences at 6 months can be expected to be maintained into the future is as yet unclear. The aim of this study was therefore to compare the long-term PROMs of those patients receiving UKA and TKA. These findings will provide important evidence that, along with the existing literature on their revision rates, can inform clinical decision-making when faced with the choice between the alternative procedures.

## Materials and methods

Patient-reported outcomes were compared following TKA, reported in the Knee Arthroplasty Trial (KAT) [35], and UKA, recorded for a cohort of patients who received a minimally invasive medial UKA [36, 37]. Propensity score matching was used to identify comparable patients based on their observed characteristics. OKS, its pain and function component scores, and EQ-5D (estimated by a mapping algorithm based on OKS) were compared for the matched patients, and mixed-effects regressions for repeated measures were used to examine the effect of surgical choice and patient characteristics on PROMs.

### Setting and participants

Data were obtained from two prospective cohorts of patients receiving TKA or UKA in the United Kingdom (UK) in which the authors of this study were involved.

KAT was a partial factorial, pragmatic, multicentre randomised controlled trial which considered whether or not to resurface the patella during TKA, whether to use a fixed or a mobile tibial bearing, and whether to use modular or all-polyethylene tibial components [35]. Patients were eligible for inclusion in the trial if a decision had been made to have primary knee arthroplasty surgery, but no particular type of operation was clearly indicated, and were randomised between July 1999 and January 2003. While UKA was included in the original design of the trial, this arm was terminated early due to the development of the minimally invasive technique for implanting unicompartmental arthroplasties [35], and these patients were excluded from this analysis. No significant clinical differences were found between the remaining patient groups, and so these patients were pooled in this analysis to provide an indication of outcomes following total knee arthroplasty.

A separate observational cohort provided evidence for outcomes following UKA [36, 37]. These patients received the first 1000 phase 3 Oxford medial unicompartmental knee arthroplasties using a minimally invasive technique and met the recommended indications for the procedure [18]. These procedures were performed by two surgeons with patients followed prospectively in a dedicated research clinic run by independent physiotherapists. A total of 818 patients received these arthroplasties with 636 unilateral and 182 bilateral (22 of which were undertaken simultaneously), with knees implanted between June 1998 and March 2009.

The index operation for all patients was a primary knee arthroplasty, either unicompartmental or total. Patients were included in the analysis if they had OKS recorded pre-operatively and at least once in the 10 years following their procedure. Outcomes were censored from when patients were lost to follow-up, had a revision or died.

### Patient-reported outcome measures and data collection

The Oxford Knee Score (OKS) is a validated questionnaire specifically developed to assess patients’ pain and function status after knee arthroplasty [11]. The questionnaire consists of 12 questions, each with five responses on a Likert scale and scored from 0 (most severe pain or limited function) to 4 (no pain or functional limitation). An overall score ranges from 0 to 48, with 48 being the best [34]. OKS can be disaggregated into a ‘OKS pain component’ and a ‘OKS functional component’ [19], with the pain component informed by seven of the questions and the functional component based on the remaining five. The OKS pain component ranges from 0 to 28, while the OKS functional component ranges from 0 to 20, with higher sores indicating less pain and better function, respectively. An overall OKS of above 41 can be considered ‘excellent’, above 34–41 as ‘good’, above 27–34 as ‘fair’, while 27 or below is a ‘poor’ score [23]. Meanwhile, the minimally clinically important change in OKS for a patient is 4 points [5].

Within the KAT study, OKS was reported by patients via postal questionnaires prior to surgery, three months after the operation and annually thereafter. Following a postal reminder, any patients who had not returned the questionnaire were contacted by telephone and offered the option of completing the questionnaire over the telephone. Similarly, the Oxford UKA cohort was sent OKS questionnaires pre- and post-surgery, and if they did not return a questionnaire, they were contacted by telephone and were able to complete the questionnaire over the phone.

EQ-5D is a widely used generic measure of health-related quality of life consisting of five questions on mobility, self-care, pain/discomfort, usual activities and anxiety/depression. Each question had three response levels with health-state preferences, or utilities, derived for the UK population [13]. Utilities indicate the relative preference that individuals associate with different health states and range from −0.5 to 1, with one representing perfect health, zero indicating death and negative values denoting health states worse than death. The minimally clinically important change in EQ-5D is 0.074 [40].

EQ-5D was not collected for the Oxford UKA cohort and was therefore estimated based on OKS responses, with individuals’ responses to each OKS question used to predict their EQ-5D score via a mapping algorithm [9]. While EQ-5D was collected in KAT alongside OKS, to provide a fair comparison, EQ-5D scores were also mapped from OKS responses for KAT patients.

### Confounders

Age at operation and gender were collected for both KAT and the UKA patients. As the association of age on outcome was nonlinear, we considered age according to the following categories used in the randomisation process in KAT study: <60 years; 60–69 years; 70–79 years and 80 years or older.

### Ethical approval

KAT received ethics approval from the Multicentre Research Ethics Committee for Scotland (ref: MREC/98/0/100). Ethical approval for the follow-up of UKA patients was not required under NHS research governance arrangements.

### Statistical methods

Patients receiving a TKA in the KAT trial were expected to differ to those in the UKA cohort thus making any unadjusted comparison between the groups ill-advised. Propensity score matching was therefore used to control for potential selection bias. The matching was generated by a logistic regression with age at procedure, gender, the Oxford Knee Score pain and function components and EQ-5D at baseline used as predictors of treatment choice. One-to-one nearest neighbour matching without replacement was then used, with a caliper of 0.2 SDs specified within which controls could be drawn [3]. Patient characteristics were compared before and after matching, with standardised mean differences used to assess the success of matching (a standardised mean difference of <0.1 indicates a negligible difference in the mean of a covariate between treatment groups) [2].

Overall OKS, its pain component, its functional component and mapped EQ-5D were compared for the two propensity score-matched treatment groups. Mean scores were estimated for each year, and bias-corrected and accelerated (BCa) confidence intervals were estimated through 2000 bootstraps.

Mixed-effects regressions for repeated measures were used to estimate the effect of treatment choice on outcomes over 10 years for the propensity score-matched patients, with random intercepts and fixed slope specified. This method takes into account incomplete follow-up data, without any imputation of missing values, and provides valid estimates of treatment effects under the assumption that such data are missing at random. Linear regressions were used with OKS, the OKS pain component, the OKS functional component and mapped EQ-5D as outcomes of interest. Logistic regressions were used to test for the effect of surgical choice on what could be considered as a successful outcome. Regressions were estimated for whether post-operative OKS was ‘excellent’ (>41), ‘excellent’ or ‘good’ (>34), or ‘excellent’, ‘good’ or ‘fair’ (>27) [23], whether the difference between post-operative OKS and EQ-5D scores exceeded or was equal to the minimally clinically important difference (4 points for OKS and 0.074 for EQ-5D) [5, 40] and whether these scores were the same or improved. Each model adjusted for age at operation, gender and baseline PROMs. As baseline PROMs were highly correlated, only the baseline score for the outcome of interest in each model was included. The merit of including interaction terms was tested through a comparison, using ANOVA, of models with and without interaction terms. Statistical significance was set at 5% (*p* < 0.05).

Due to inherent differences in study designs, the proportion of missing data could be expected to differ for KAT and the Oxford UKA cohort. If outcomes of those with missing data and those excluded from the analysis differed from those who were included, then findings would be biased. Therefore, the characteristics and outcomes of those eligible for inclusion in the analysis were compared with those who were excluded.

Except for the mapping of Oxford Knee Scores to EQ-5D, which was done in Stata 12 using the oks2eq command [38], data analysis was undertaken in R 3.3.0. Propensity score matching was implemented using the MatchIt package [20], and mixed-effect regressions were implemented using the Lme4 package with the sjPlot used to summarise results [4, 32].

### Sensitivity analysis

Two sensitivity analyses were undertaken to assess how robust the results of the study were to changes in analytic decisions. First, the analysis was repeated using an alternative approach to propensity score matching where the requirement that controls could only be drawn from a caliper of 0.2 SDs was removed. Second, as knee arthroplasties in the Oxford UKA cohort were performed by two experienced surgeons, the analysis was re-run with only knee arthroplasties in KAT done by a consultant or an associate specialist included.

### Sample size consideration

Data collection was not designed for this analysis but, rather, data were collected to inform independent considerations of alternative methods of TKA and patient outcomes following minimally invasive UKA. This analysis made use of all available data which satisfied the inclusion criteria.

## Results

### Study participants

**Fig. 1:**
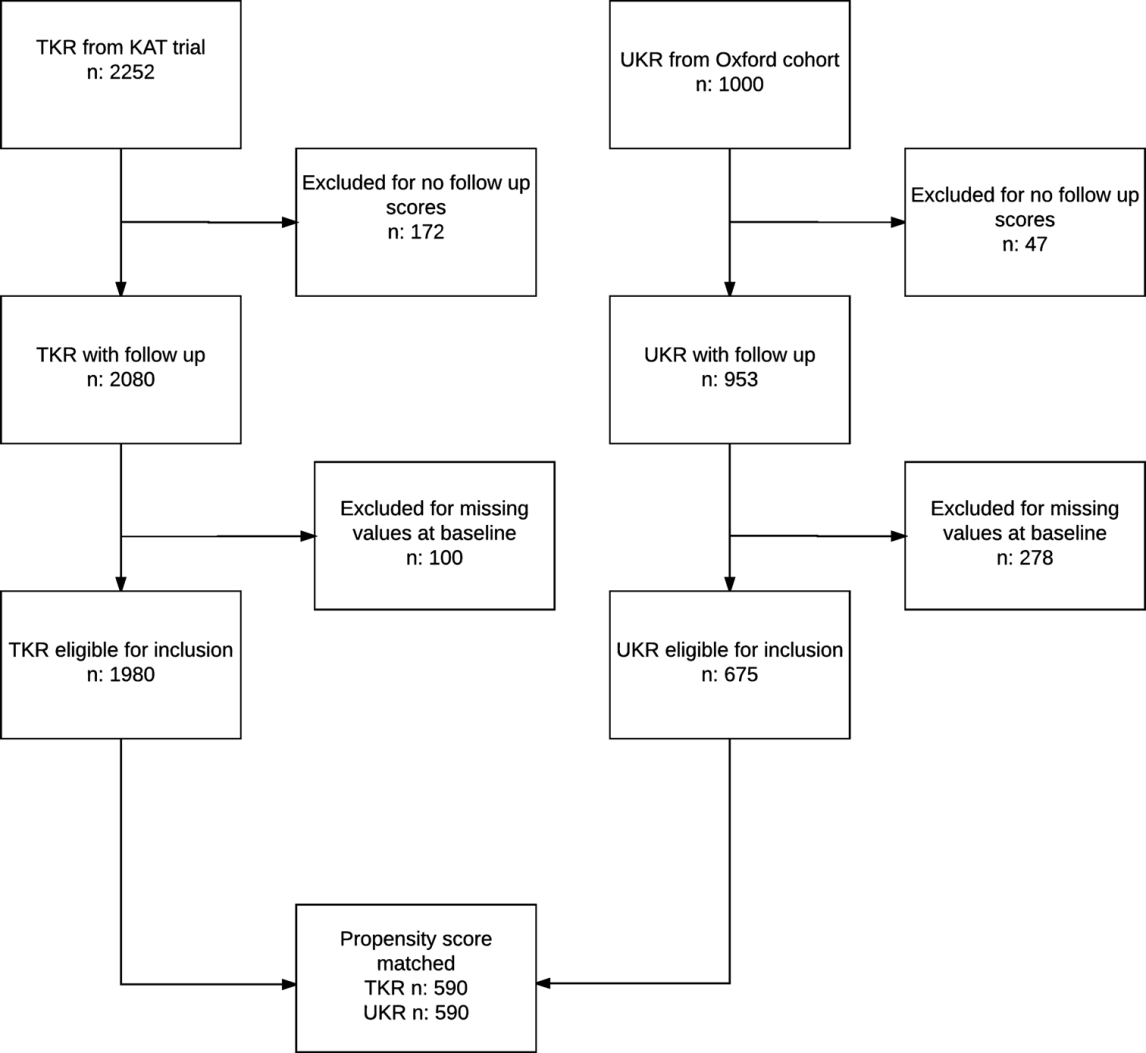
Flow chart of study inclusion

A flow chart detailing further inclusion/exclusion of patients is presented in Fig. 1, with the characteristics of those eligible and ineligible for inclusion are described in Appendix 1 as Electronic Supplementary. Given the similarity in outcomes between those eligible and ineligible, imputation of missing data was deemed to be unnecessary. Prior to propensity score matching, patients receiving UKA were more likely to be younger, male and to have better health status than those receiving TKA. After propensity score matching, with 590 UKAs matched to 590 TKAs, these characteristics were well balanced with standardised mean differences <0.1. Patient characteristics before and after matching are detailed in Table 1. Twenty-four (4.1%) TKAs and 23 (3.9%) UKAs were revised over the 10 years of follow-up, and any patient-reported outcomes following revision were censored from the analysis.

**Table 1:** Patient characteristics before and after matching

	Before matching		Standardised mean difference	After matching		Standardised mean difference
	TKA	UKA		TKA	UKA	
*N*	1980	675		590	590	
Age (mean, SD)	70.4 (8.2)	66.8 (9.6)	0.40	67.59 (8.37)	67.66 (9.0)	0.01
Gender: male (*n*, %)	864 (44%)	350 (52%)	0.17	292 (49%)	293 (50%)	0.00
Pre-op OKS pain component (mean, SD)	9.61 (4.6)	12.63 (5.2)	0.62	11.56 (5.2)	11.82 (4.8)	0.05
Pre-op OKS function component (mean, SD)	8.57 (3.5)	11.95 (4.0)	0.89	10.9 (3.7)	11.19 (3.7)	0.08
Pre-op OKS (mean, SD)	18.18 (7.5)	24.59 (8.7)	0.79	22.46 (8.3)	23.03 (8.0)	0.07
Pre-op EQ-5D (median, IQ)	0.4 (0.2–0.6)	0.61 (0.4–0.7)	0.62	0.57 (0.3–0.7)	0.58 (0.3–0.7)	0.07

Standardised mean difference compares the difference in means in units of the pooled standard deviation

*TKA* total knee arthroplasty, *UKA* unicompartmental knee arthroplasty, *SD* standard deviation, *IQ* interquartile range

### Patient-reported outcomes

At one year following primary surgery, patients who received an UKA reported a mean OKS of 40.3 (95% CI 39.5–41.0), while those who received a TKA reported a mean OKS of 35.9 (95% CI 35.0–37.6). Differences persisted over the period of follow-up with those patients who received a UKA reporting a mean OKS of 39.2 (95% CI 38.1–40.2) after 10 years, while those who received a TKA reported a mean OKS of 35.9 (95% CI 34.6–37.0). Appendix 2 as Electronic Supplementary shows the mean values of observed OKS, its pain and function components, and the mapped EQ-5D for propensity score-matched UKA and TKA patients with their corresponding bootstrapped confidence intervals.

Reported scores were better for UKA than TKA for both the OKS pain and function components, where higher scores indicate less pain and better function, respectively. At one year following surgery, patients with UKA reported on average 23.8 (23.3–24.2) for the pain component and 16.5 (16.2–16.7) for the function component, while TKA patients reported 22.0 (21.5–22.5) for the pain component and 14.1 (13.8–14.5) for the function component. After 10 years, scores for UKA patients were 23.6 (22.8–24.3) for the pain component and 15.3 (14.7–15.9) for the function component, while TKA patients reported 22.2 (21.5–22.9) and 13.7 (13.2–14.3).

EQ-5D, mapped from these OKS scores, was estimated to be on average 0.82 (0.80–0.83) for UKA patients and 0.74 (0.72–0.76) for TKA patients at the first year post-surgery. After 10 years, EQ-5D for UKA patients was 0.78 (0.76–0.8) and 0.74 (0.72–0.76) for TKA.

### Factors associated with patient-reported outcomes

**Fig. 2:**
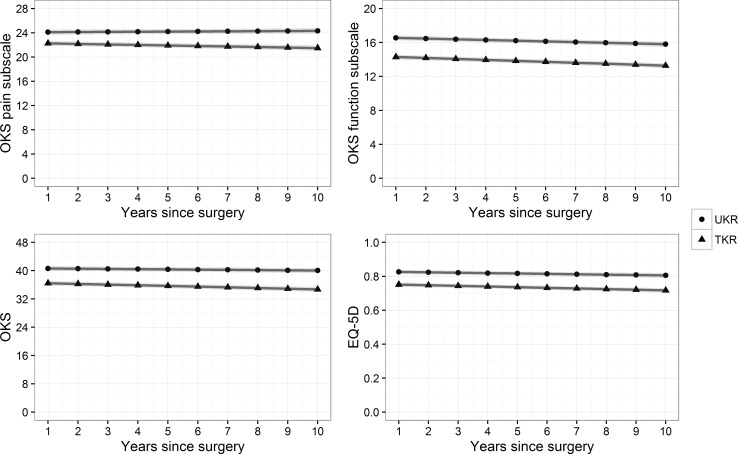
Marginal effect of treatment and time on patient-reported outcomes from mixed-effects regressions. A higher score indicates better outcomes for each measure. Yearly point estimates with *shaded areas* reflecting 95% confidence intervals

Table 2 summarises the results from the mixed-effects linear regressions of OKS, its pain and function components, and mapped EQ-5D scores. A statistically significant interaction was found between surgery and years since surgery for the linear regressions (Fig. 2). The provision of UKA rather than TKA was associated with statistically significant increased scores for overall OKS, both of its pain and function components, and EQ-5D. While all scores for TKA patients are expected to fall over time, UKA scores are expected to fall to a lesser degree or, in the case of the OKS pain component, to improve. Based on regressions of PROMs recorded for year 1 (Appendix 4 as Electronic Supplementary), the provision of UKA rather than TKA was associated with an additional 4.1 points for OKS (*p* < 0.05), with an additional 1.9 (*p* < 0.05) points in the pain component and 1.6 (*p* < 0.05) for the function component, while EQ-5D was estimated to be higher by 0.06 (*p* < 0.01). At year 10, OKS for UKA was expected to be 4.5 (*p* < 0.05) higher than for TKA, with a difference of 1.7 (*p* < 0.05) for the pain component and 2.1 (*p* < 0.05) for the function component, and EQ-5D was expected to be 0.07 (*p* < 0.05) higher for UKA. Table 3 details the results from the mixed-effects logistic regressions on the effect of surgical choice on patient-reported outcomes. The use of UKA was associated with greater odds of achieving a successful outcome.

**Table 2:** Effect of surgical choice and years since surgery on patient-reported outcome scores over 10 years following surgery

		OKS pain component (0 to 28)	OKS function component (0 to 20)	OKS (0 to 48)	EQ-5D (−0.59 to 1)
Surgery (UKA)	Estimate (95% CI)	1.7 (1.1 to 2.4)*	2.2 (1.8 to 2.7)*	4.0 (3.1 to 5.0)*	0.07 (0.05 to 0.09)*
Years since surgery	Estimate (95% CI)	−0.1 (−0.1 to −0.1)*	−0.1 (−0.1 to −0.1)*	−0.2 (−0.3 to −0.1)*	−0.00 (−0.01 to −0.00)*
Surgery (UKA)* Years since surgery	Estimate (95% CI)	0.1 (0.1 to 0.2)*	0.0 (−0.0 to 0.1)^n.s.^	0.1 (0.0 to 0.2)*	0.00 (−0.00 to 0.00)^n.s^

Estimated effect of surgical choice from mixed-effects linear regressions controlling for years since surgery, age group, gender and pre-operative scores (Appendix 3 as Electronic Supplementary). An interaction term between type of surgery and years since surgery was included in the model. The estimated effect associated with surgical choice is a product of the combination of these three coefficient estimates

*n.s.* not significant

* *p* < 0.05

**Table 3:** Effect of surgical choice on the odds of attaining successful patient-reported outcomes over 10 years following surgery

	Surgery (UKA) OR (95% CI)
*OKS categories*	
‘Excellent’ (>41)	4.6 (3.2–6.5)*
‘Excellent’ or ‘good’ (>34)	3.8 (2.5–5.9)*
‘Excellent’, ‘good’ or ‘fair’ (>27)	3.5 (2.0–6.0)*
*Change in OKS*	
Achieve MCID (≥4)	2.9 (1.8–4.7)*
No change or improvement (≥0)	3.0 (1.7–5.5)*
*Change in EQ*-*5D*	
Achieve MCID (≥0.074)	2.5 (1.6–3.8)*
No change or improvement (≥0)	2.1 (1.4–3.6)*

*OR* odds ratio (OR) based on mixed-effects logistic regression controlling for years since surgery, age group, gender and pre-operative scores (Appendix 5 as Electronic Supplementary). OKS categories are based on absolute scores, while change in OKS and EQ-5D compares patients’ post-operative scores with their pre-operative score

*MCID* Minimally clinically important difference, *OKS* Oxford knee score, *UKA* unicompartmental knee arthroplasty, *CI* confidence intervals

* *p* < 0.05

A number of patient factors were included in the multivariate regressions, with the full model specifications detailed in Appendixes 3 and 5 as Electronic Supplementary. Patients aged between 60 and 70 were associated with better OKS and EQ-5D for both UKA and TKA than those aged below 60 (−1.5, n.s., and −0.03, *p* < 0.05, respectively), those aged 70–80 (−1.4 and −0.03, *p* < 0.05), and those older than 80 (−0.8 and −0.03, n.s.). For both UKA and TKA, male gender was associated with a better OKS function component (0.2), but a worse OKS pain component (−0.3) and overall OKS (−0.2) and no difference in EQ-5D, although none of these differences were found to be statistically significant. Better pre-operative scores were associated with better post-operative scores for both OKS (0.4, *p* < 0.05), including the pain (0.3, *p* < 0.05) and function (0.5, *p* < 0.05) components, and EQ-5D (0.27, *p* < 0.05). These indicate that, for example, a one-point higher score for OKS pre-operatively is associated with on average a 0.4-point higher score post-operatively.

### Sensitivity analysis

Propensity score matching without the requirement that controls could only be drawn from a caliper of 0.2 SDs allowed all 678 eligible UKAs to be included in the analysis. Matched UKA and TKA patients were not well balanced with only gender and age having a standardised mean difference of <0.1 (see Appendix 6, Table A.6.1 as Electronic Supplementary). The results from the analysis were similar to those in the primary analysis (see Appendix 6, Table A.6.2 as Electronic Supplementary).

Only including those knee arthroplasties undertaken by a consultant or associate specialist in KAT led to 1428 KAT patients being eligible for inclusion (see Appendix 7, Table A.7.1 as Electronic Supplementary). After repeating the propensity score matching, this additional restriction did not have any substantial effect on the results of the analysis with the provision of UKA associated with similar improvements in PROMs as found in the base case analysis (see Appendix 7, Table A.7.2 as Electronic Supplementary).

## Discussion

The most important finding of the study is that those patients who received one of the first 1000 phase 3 Oxford medial unicompartmental knee arthroplasties using a minimally invasive technique have reported better patient-reported outcomes than comparable patients who received a total knee arthroplasty as part of the Knee Arthroplasty Trial. These differences in outcomes were sustained across 10 years following the procedures. While the differences in observed scores decreased over time, after controlling for age, gender and baseline scores using multivariate regression models, the differences in outcomes between UKA and TKA are in fact estimated to grow over time. While all scores for TKA patients are expected to fall, UKA scores are expected to fall to a lesser degree or, in the case of the OKS pain component, to improve.

The difference in OKS is driven by greater improvements in both its pain and function components. As would be expected, given the effects of normal ageing, OKS function component scores fall over time [21, 41]. However, these reductions in OKS function component scores are more pronounced for TKA patients. In addition, OKS pain component scores, which are less influenced by the ageing process [7], diverge with UKA pain scores improving over time while those of TKA worsen. The provision of UKA rather than TKA is also associated with an increased likelihood of a successful outcome, with receiving UKA more likely to achieve the minimally clinically important improvements in both OKS and EQ-5D, and an ‘excellent’ post-operative OKS.

The difference in outcomes estimated here exceeds that observed for routinely collected NHS PROMs after six months, where UKA was associated with an adjusted mean difference of 1.5 for OKS and 0.02 for EQ-5D, and odds ratios of 1.3 for achieving a minimally clinically important difference in OKS and 1.6 for an ‘excellent’ OKS [29]. This increased treatment effect is likely due to the characteristics of the UKAs included here, which were performed by surgeons with a high usage and caseload of UKA at a high-volume centre, all of which has been found to be associated with better outcomes [6, 28, 30].

For both the UKA and TKA patients for this analysis, better pre-operative scores are associated with better post-operative OKS. This is consistent with previous research on those receiving both UKA and TKA [21, 22, 27].

The estimated effects of gender on PROMs are mixed with no statistically significant differences found. Previous findings for the effect of gender are also mixed as while being male has been found to be associated with a lower OKS following TKA, with the difference attributed to the function component [22], being male has been found to be associated with better scores following UKA [27].

PROMs were found in our analysis to be better for those aged between 60 and 70, with both younger and older patients reporting worse outcomes. Previous estimates for the effect of age on outcomes have been mixed, with one study finding no relationship [17], others finding that older age to be related with poorer outcomes [14, 16, 22, 24, 31], while two others found outcomes to peak among those aged between 60 and 80 and for those aged 75, respectively [21, 27]. The findings from this analysis are in line with these latter two studies.

This analysis is informed by evidence from two patient cohorts, with KAT providing evidence on outcomes following TKA, while a cohort of 1000 patients who received a minimally invasive phase 3 Oxford medial UKA provides evidence for UKA. Both studies were rigorously undertaken, and a number of publications have reported results from them independently [35–37]. PROMs were reported in each study over 10 years following knee arthroplasty, and synthesising the studies has allowed us to provide the first, to our knowledge, comparison of long-term patient-reported outcome measures following UKA and TKA.

Patient characteristics differed across the studies, with those in the UKA cohort more likely to be younger, male and to report better pre-operative PROMs. This is consistent with current provision in the NHS [26, 29]. Propensity score matching was used here to achieve similar distributions in observed baseline covariates so as to minimise potential confounding. However, while differences in observed patient characteristics were minimised, differences may have remained in unobserved characteristics. In particular, patient comorbidities and pre-operative radiographs were not included and although both can be expected to be correlated with baseline PROMs, and so controlled for to some degree, imbalances in these may well remain. Moreover, differences in health care not associated with treatment choice, such as access to physiotherapy, may have existed between the groups.

As well as differences in patient characteristics, differences in the characteristics of the surgeons in the two studies could also lead to differences in treatment effects limiting the generalisability of the results. Knee arthroplasties for the Oxford UKA cohort were performed by two experienced surgeons, while those done in KAT were performed by surgeons with a range of experience. Limiting our selection criteria to include only TKAs undertaken by a consultant or associate specialist in KAT did not have any substantial effect on the results of the analysis. It can be expected, however, that if the UKA cohort could be expanded to include low usage surgeons, UKA outcomes would fall [30].

The studies used to inform this analysis also differed in their proportions of missing data. In particular, while 6% of KAT patients were excluded on the basis of missing pre-operative scores, 28% of the Oxford UKA cohort were. These differing rates could lead to bias if missingness was not completely at random. If, for example, those excluded were more likely to have worse outcomes, then the estimated effect of UKA would be exaggerated. However, it was observed that excluded patients had similar characteristics at baseline and similar outcomes than included patients. Based on this finding, missing pre-operative OKS are not expected to lead to bias, although they can be expected to have widened the uncertainty surrounding estimates of the effects of patient characteristics and type of surgery [39].

## Conclusion

Minimally invasive UKAs performed on patients with the appropriate indications resulted in better patient-reported health outcomes than total knee arthroplasties performed on similar patients. Differences in patient-reported outcomes were maintained over 10 years demonstrating the long-term impact of the choice of surgical procedure on health outcomes.

## Electronic supplementary material

Below is the link to the electronic supplementary material.

Supplementary material 1 (PDF 728 kb)
