# Supplementary material for: Ten-year patient-reported outcomes following total and minimally invasive unicompartmental knee arthroplasty: a propensity score-matched cohort analysis
Source: Knee Surg Sports Traumatol Arthrosc. 2016 Dec 29;26(5):1455–64. doi: 10.1007/s00167-016-4404-7 (PMC5907625; doi:10.1007/s00167-016-4404-7)
Supplement: Supplementary file 1 — Supplementary material 1 (PDF 728 kb) [file 167_2016_4404_MOESM1_ESM.pdf]

APPENDIX 1

Table A.1.1. Comparison of ineligible and eligible TKR

TKR	All	Missing	Excluded	Eligible	P value
N	2,252	2252	272	1,980	
Age (mean, SD)	70.59 (8.31)	0 (0%)	71.93 (9.17)	70.40 (8.17)	0.009*
Gender (male, %)	981 (44%)	0 (0%)	117 (43%)	864 (44%)	0.846†
Pre-op OKS (mean, SD)	17.97 (7.53)	121 (5.37%)	15.21 (7.34)	18.18 (7.50)	<0.001*
Post-op OKS year 1 (median, IQR)	36 (28 to 42)	558 (24.78%)	33.5 (25.25 to 42)	36 (28 to 42)	0.276‡
Post-op OKS year 2 (median, IQR)	38 (28 to 43)	707 (31.39%)	38 (28.25 to 42)	38 (28 to 43)	0.78‡
Post-op OKS year 3 (median, IQR)	37 (28 to 43)	643 (28.55%)	38 (30 to 44)	37 (28 to 43)	0.482‡
Post-op OKS year 4 (median, IQR)	37 (27 to 43)	655 (29.09%)	39.0 (29 to 44)	36 (27 to 43)	0.219‡
Post-op OKS year 5 (median, IQR)	37 (28 to 43)	741 (32.9%)	39 (28 to 44)	37 (28 to 43)	0.296‡
Post-op OKS year 6 (median, IQR)	37 (28 to 43)	838 (37.21%)	39 (31 to 43)	37 (28 to 43)	0.343‡
Post-op OKS year 7 (median, IQR)	37 (27 to 43)	912 (40.5%)	37 (30 to 41.75)	37 (27 to 43)	0.766‡
Post-op OKS year 8 (median, IQR)	36 (27 to 43)	989 (43.92%)	37 (28 to 41)	36 (27 to 43)	0.78‡
Post-op OKS year 9 (median, IQR)	36 (27 to 43)	1105 (49.07%)	36.0 (30 to 42)	36 (27 to 43)	0.528‡
Post-op OKS year 10 (median, IQR)	36 (26 to 43)	1316 (58.44%)	35 (27 to 42)	36.0 (26 to 43)	0.684‡
Pre-op EQ-5D (median, IQR)	0.41 (0.16 to 0.59)	121 (5.37%)	0.23 (0.07 to 0.51)	0.43 (0.16 to 0.6)	<0.001‡
Post-op EQ-5D year 1 (median, IQR)	0.73 (0.62 to 0.86)	558 (24.78%)	0.72 (0.59 to 0.85)	0.73 (0.62 to 0.86)	0.379‡
Post-op EQ-5D year 2 (median, IQR)	0.75 (0.62 to 0.88)	707 (31.39%)	0.74 (0.62 to 0.86)	0.75 (0.62 to 0.88)	0.558‡
Post-op EQ-5D year 3 (median, IQR)	0.75 (0.62 to 0.88)	643 (28.55%)	0.77 (0.63 to 0.89)	0.75 (0.62 to 0.88)	0.646‡
Post-op EQ-5D year 4 (median, IQR)	0.73 (0.61 to 0.88)	655 (29.09%)	0.75 (0.65 to 0.91)	0.73 (0.61 to 0.88)	0.262‡
Post-op EQ-5D year 5 (median, IQR)	0.74 (0.61 to 0.89)	741 (32.9%)	0.76 (0.62 to 0.92)	0.73 (0.61 to 0.88)	0.239‡
Post-op EQ-5D year 6 (median, IQR)	0.74 (0.61 to 0.88)	838 (37.21%)	0.75 (0.63 to 0.89)	0.74 (0.61 to 0.88)	0.628‡
Post-op EQ-5D year 7 (median, IQR)	0.73 (0.61 to 0.88)	912 (40.5%)	0.74 (0.64 to 0.91)	0.73 (0.61 to 0.88)	0.418‡
Post-op EQ-5D year 8 (median, IQR)	0.71 (0.6 to 0.87)	989 (43.92%)	0.72 (0.64 to 0.83)	0.71 (0.6 to 0.87)	0.919‡
Post-op EQ-5D year 9 (median, IQR)	0.71 (0.59 to 0.87)	1105 (49.07%)	0.72 (0.63 to 0.86)	0.71 (0.59 to 0.87)	0.741‡
Post-op EQ-5D year 10 (median, IQR)	0.71 (0.59 to 0.87)	1316 (58.44%)	0.69 (0.58 to 0.84)	0.71 (0.59 to 0.87)	0.626‡

TKR: total knee replacement. *t-test †Chi 2 ‡Wilcoxon rank-sum test.

Table A.1.2. Comparison of ineligible and eligible UKR

UKR	All	Missing	Excluded	Eligible	P value
N	1,000	1000	325	675	
Age (mean, SD)	66.59 (9.63)	0 (0%)	66.11 (9.78)	66.82 (9.56)	0.276*
Gender (male, %)	513 (51%)	0 (0%)	163 (50%)	350 (52%)	0.615†
Pre-op OKS (mean, SD)	24.68 (8.77)	308 (30.8%)	28.35 (11.28)	24.59 (8.69)	0.19*
Post-op OKS year 1 (median, IQR)	43 (38 to 46)	237 (23.7%)	43.0 (38 to 46)	43 (38 to 46)	0.756‡
Post-op OKS year 2 (median, IQR)	44 (38 to 46)	512 (51.2%)	44 (37.5 to 46)	43 (38 to 46)	0.895‡
Post-op OKS year 3 (median, IQR)	44 (39 to 47)	691 (69.1%)	44 (38.75 to 47)	44 (39 to 47)	0.525‡
Post-op OKS year 4 (median, IQR)	44 (39 to 47)	729 (72.9%)	44.5 (39 to 47)	44 (40 to 47)	0.816‡
Post-op OKS year 5 (median, IQR)	44 (39 to 47)	190 (19%)	44 (38 to 46.5)	44 (39 to 47)	0.53‡
Post-op OKS year 6 (median, IQR)	44 (38 to 47)	698 (69.8%)	43 (35.25 to 46)	44 (39 to 47)	0.122‡
Post-op OKS year 7 (median, IQR)	44 (38 to 47)	365 (36.5%)	44 (38 to 47)	44 (39 to 47)	0.63‡
Post-op OKS year 8 (median, IQR)	44 (36 to 47)	817 (81.7%)	45 (37 to 47)	44 (35 to 47)	0.471‡
Post-op OKS year 9 (median, IQR)	43 (34.5 to 47)	825 (82.5%)	41.5 (35.25 to 46)	44 (34 to 47)	0.301‡
Post-op OKS year 10 (median, IQR)	43 (36 to 47)	545 (54.5%)	43 (36.5 to 47)	42.5 (35.75 to 47)	0.642‡
Pre-op EQ-5D (median, IQR)	0.61 (0.37 to 0.68)	309 (30.9%)	0.66 (0.34 to 0.71)	0.61 (0.37 to 0.68)	0.507‡
Post-op EQ-5D year 1 (median, IQR)	0.87 (0.76 to 0.93)	294 (29.4%)	0.87 (0.77 to 0.92)	0.87 (0.76 to 0.93)	0.534‡
Post-op EQ-5D year 2 (median, IQR)	0.89 (0.77 to 0.94)	560 (56%)	0.90 (0.78 to 0.94)	0.89 (0.77 to 0.94)	0.424‡
Post-op EQ-5D year 3 (median, IQR)	0.90 (0.79 to 0.94)	726 (72.6%)	0.91 (0.78 to 0.94)	0.89 (0.79 to 0.93)	0.393‡
Post-op EQ-5D year 4 (median, IQR)	0.88 (0.77 to 0.94)	802 (80.2%)	0.89 (0.78 to 0.94)	0.88 (0.77 to 0.93)	0.675‡
Post-op EQ-5D year 5 (median, IQR)	0.89 (0.77 to 0.93)	243 (24.3%)	0.88 (0.77 to 0.93)	0.89 (0.78 to 0.94)	0.451‡
Post-op EQ-5D year 6 (median, IQR)	0.88 (0.74 to 0.94)	793 (79.3%)	0.88 (0.73 to 0.93)	0.89 (0.75 to 0.94)	0.34‡
Post-op EQ-5D year 7 (median, IQR)	0.90 (0.76 to 0.94)	391 (39.1%)	0.89 (0.75 to 0.93)	0.90 (0.76 to 0.94)	0.31‡
Post-op EQ-5D year 8 (median, IQR)	0.91 (0.72 to 0.94)	847 (84.7%)	0.92 (0.75 to 0.94)	0.90 (0.71 to 0.93)	0.269‡
Post-op EQ-5D year 9 (median, IQR)	0.81 (0.68 to 0.93)	877 (87.7%)	0.82 (0.71 to 0.92)	0.80 (0.67 to 0.93)	0.919‡
Post-op EQ-5D year 10 (median, IQR)	0.84 (0.69 to 0.93)	645 (64.5%)	0.82 (0.68 to 0.93)	0.84 (0.69 to 0.94)	0.79‡

UKR: unicompartmental knee replacement. *t-test †Chi 2 ‡Wilcoxon rank-sum test.

Figure A.1.1. Oxford Knee Score of eligible and excluded patients

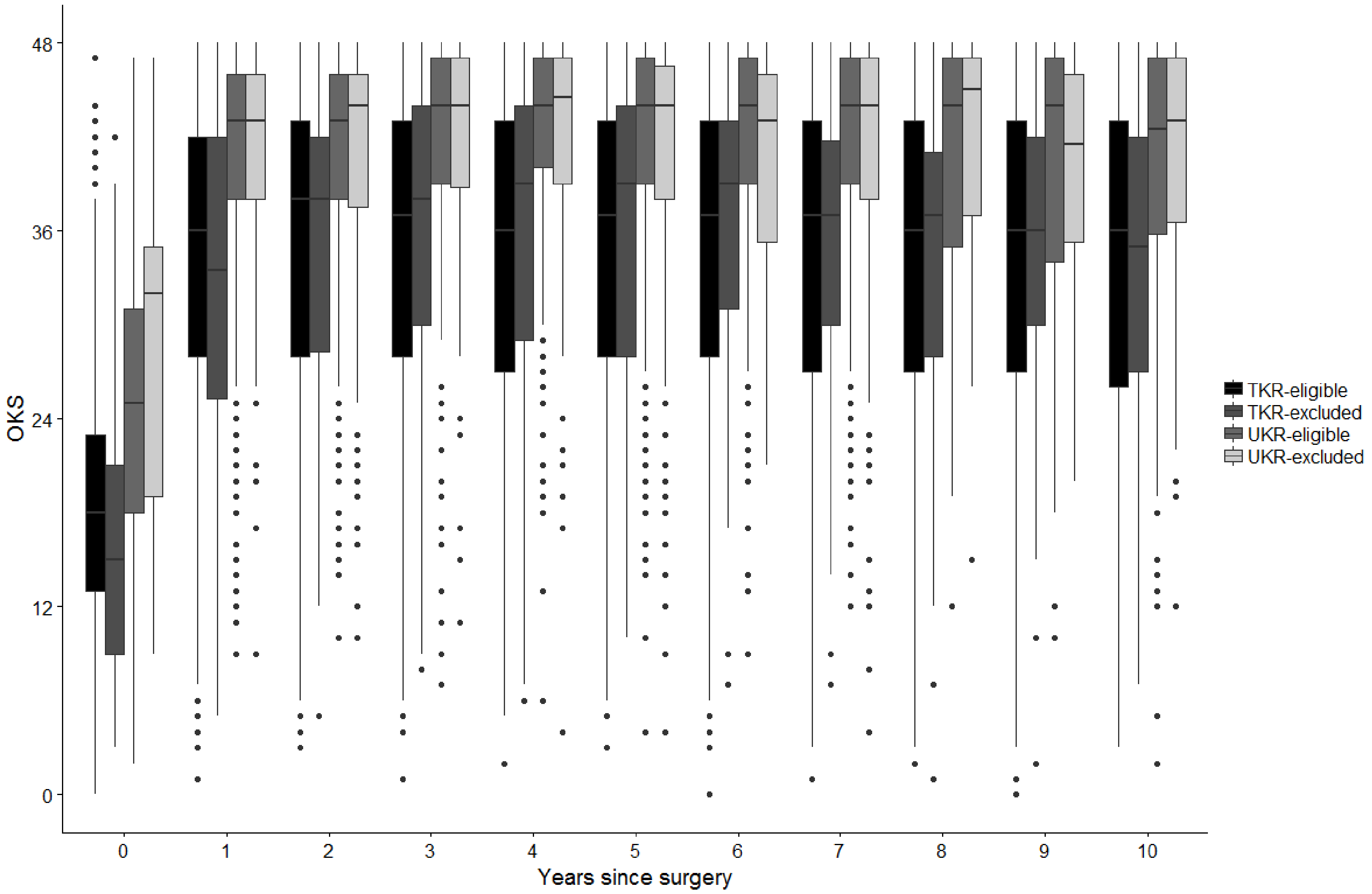

Figure A.1.2. Mapped EQ-5D of eligible and excluded patients

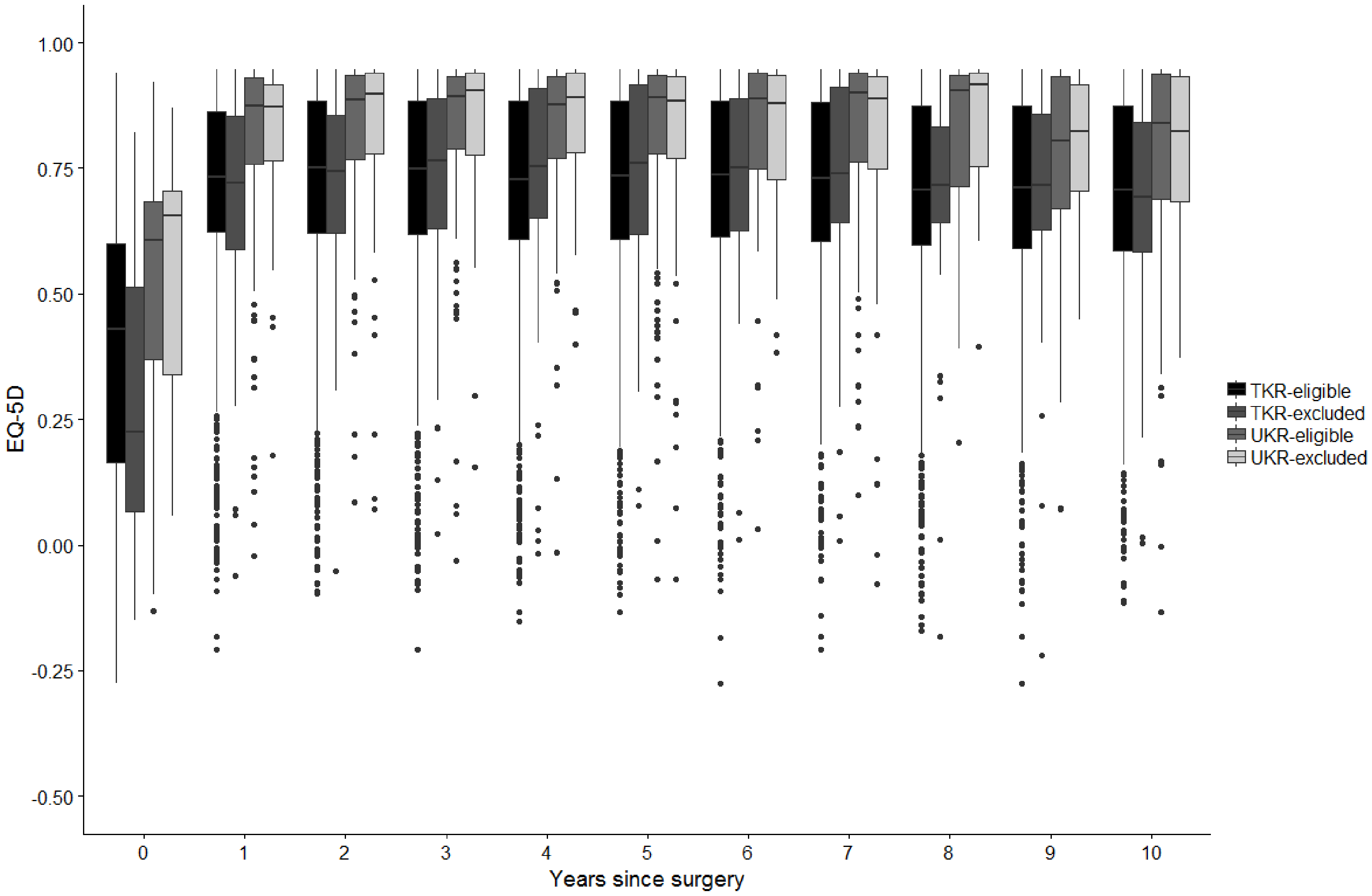

APPENDIX 2

Table A.2.1. Observed patient-reported outcomes. Mean values with 95% bootstrapped confidence intervals.

Year	OKS pain component		OKS function component		OKS		Mapped EQ-5D	
	TKR	UKR	TKR	UKR	TKR	UKR	TKR	UKR
1	22.02 (21.49 to 22.52)	23.82 (23.33 to 24.22)	14.14 (13.76 to 14.5)	16.47 (16.15 to 16.74)	35.89 (35.01 to 36.75)	40.26 (39.52 to 40.95)	0.74 (0.72 to 0.76)	0.82 (0.8 to 0.83)
2	22.73 (22.23 to 23.27)	24.27 (23.65 to 24.84)	14.34 (13.94 to 14.72)	16.5 (16.05 to 16.89)	36.83 (35.84 to 37.74)	40.62 (39.62 to 41.46)	0.76 (0.74 to 0.77)	0.83 (0.81 to 0.84)
3	22.42 (21.87 to 22.93)	24.21 (23.3 to 24.92)	14.21 (13.8 to 14.63)	16.28 (15.69 to 16.8)	36.39 (35.43 to 37.28)	40.25 (38.93 to 41.38)	0.75 (0.73 to 0.77)	0.82 (0.79 to 0.85)
4	22.33 (21.76 to 22.84)	24.17 (23.24 to 24.93)	13.94 (13.52 to 14.35)	16.32 (15.65 to 16.91)	36.06 (35.05 to 36.96)	40.94 (39.55 to 42.03)	0.74 (0.72 to 0.76)	0.82 (0.79 to 0.84)
5	22.53 (21.98 to 23.07)	24.38 (23.94 to 24.79)	14.08 (13.64 to 14.47)	16.48 (16.15 to 16.77)	36.41 (35.48 to 37.33)	41.01 (40.29 to 41.66)	0.75 (0.73 to 0.77)	0.83 (0.81 to 0.84)
6	22.55 (21.98 to 23.09)	23.92 (22.78 to 24.85)	14.52 (14.05 to 14.91)	16.33 (15.51 to 17.02)	37.03 (36.09 to 37.93)	40.64 (39.34 to 41.76)	0.76 (0.74 to 0.78)	0.81 (0.77 to 0.84)
7	22.39 (21.78 to 22.96)	24.76 (24.26 to 25.19)	14.14 (13.7 to 14.57)	16.3 (15.92 to 16.64)	36.42 (35.35 to 37.32)	40.98 (40.2 to 41.71)	0.75 (0.73 to 0.77)	0.83 (0.81 to 0.84)
8	22.41 (21.75 to 22.97)	24.05 (22.93 to 24.99)	13.8 (13.35 to 14.24)	16.19 (15.38 to 16.93)	36.05 (35.08 to 37.08)	40.32 (38.73 to 41.75)	0.74 (0.72 to 0.76)	0.81 (0.78 to 0.84)
9	22.22 (21.59 to 22.83)	23.02 (21.25 to 24.39)	13.8 (13.32 to 14.26)	15.02 (13.89 to 15.96)	35.96 (34.79 to 37.01)	39.6 (37.47 to 41.27)	0.74 (0.72 to 0.76)	0.77 (0.71 to 0.81)
10	22.21 (21.51 to 22.88)	23.62 (22.84 to 24.27)	13.74 (13.2 to 14.3)	15.31 (14.72 to 15.87)	35.87 (34.64 to 37.04)	39.22 (38.08 to 40.19)	0.74 (0.72 to 0.76)	0.78 (0.76 to 0.8)

Figure A.2.1. Observed patient-reported outcomes. Mean scores with bootstrapped confidence Intervals. A higher score indicates a better health for each outcome measure.

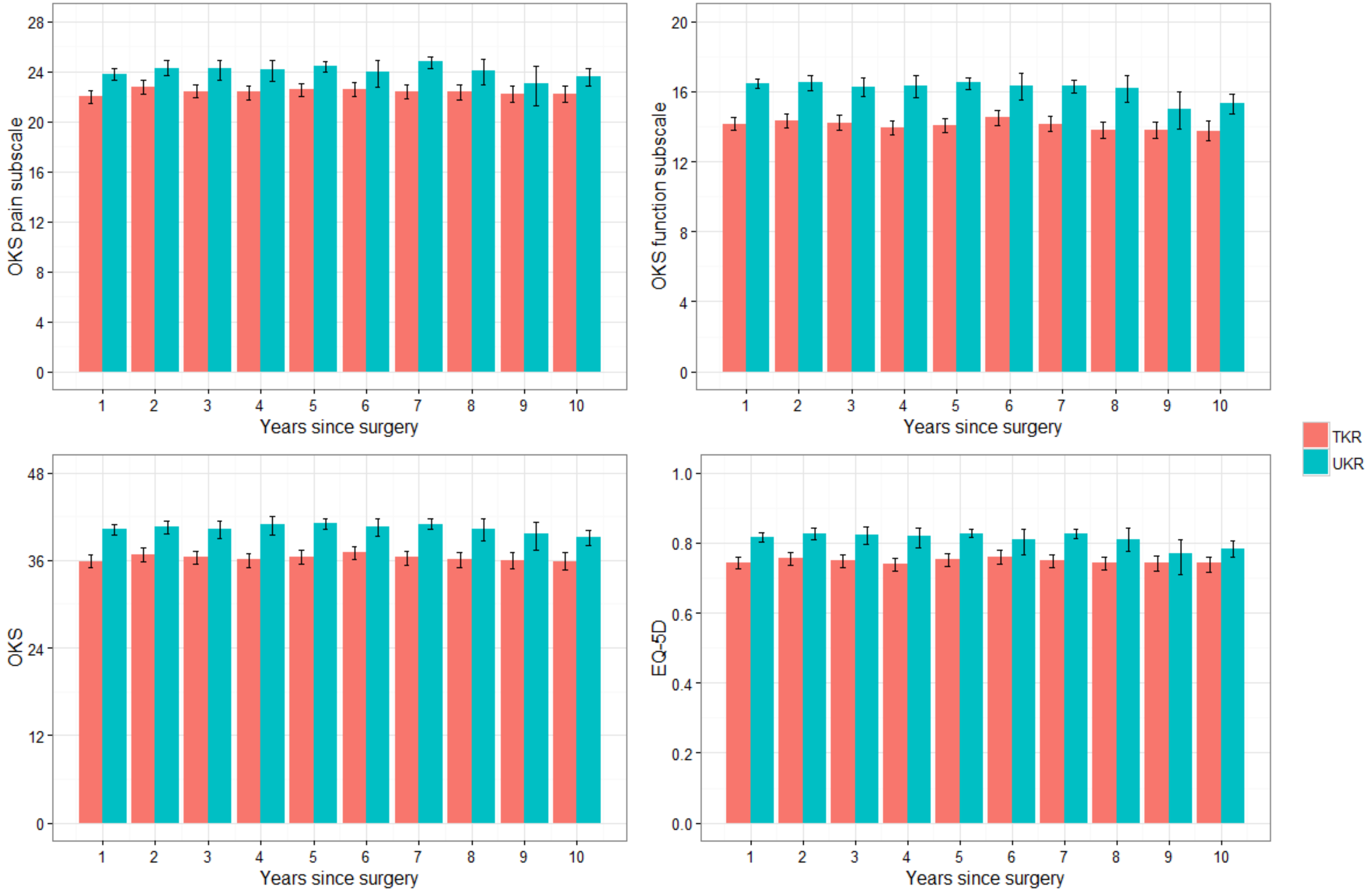

Figure A.2.2. Box plot of observed Oxford Knee Score of matched patients

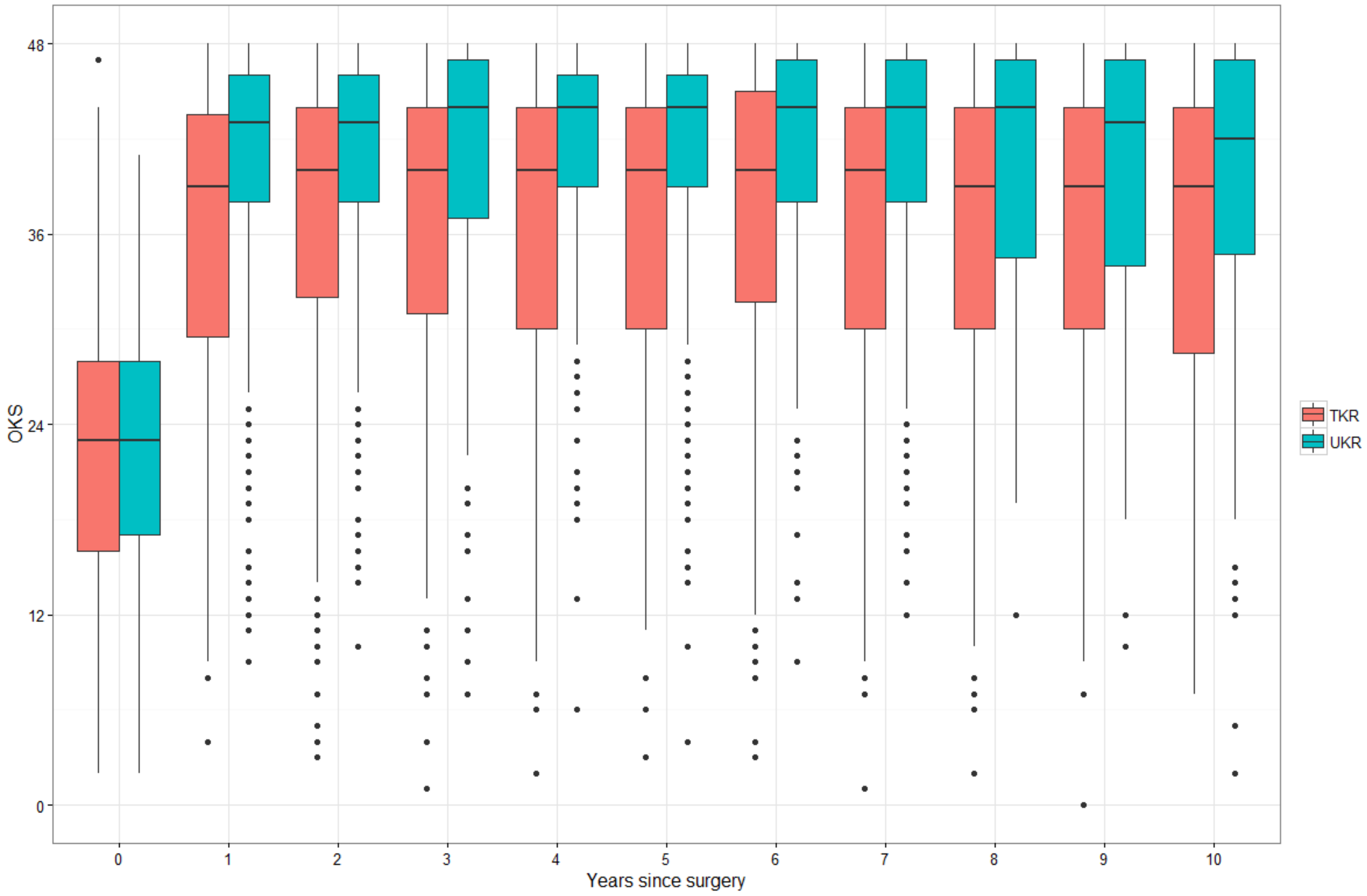

Figure A.2.3. Box plot of observed EQ-5D of matched patients

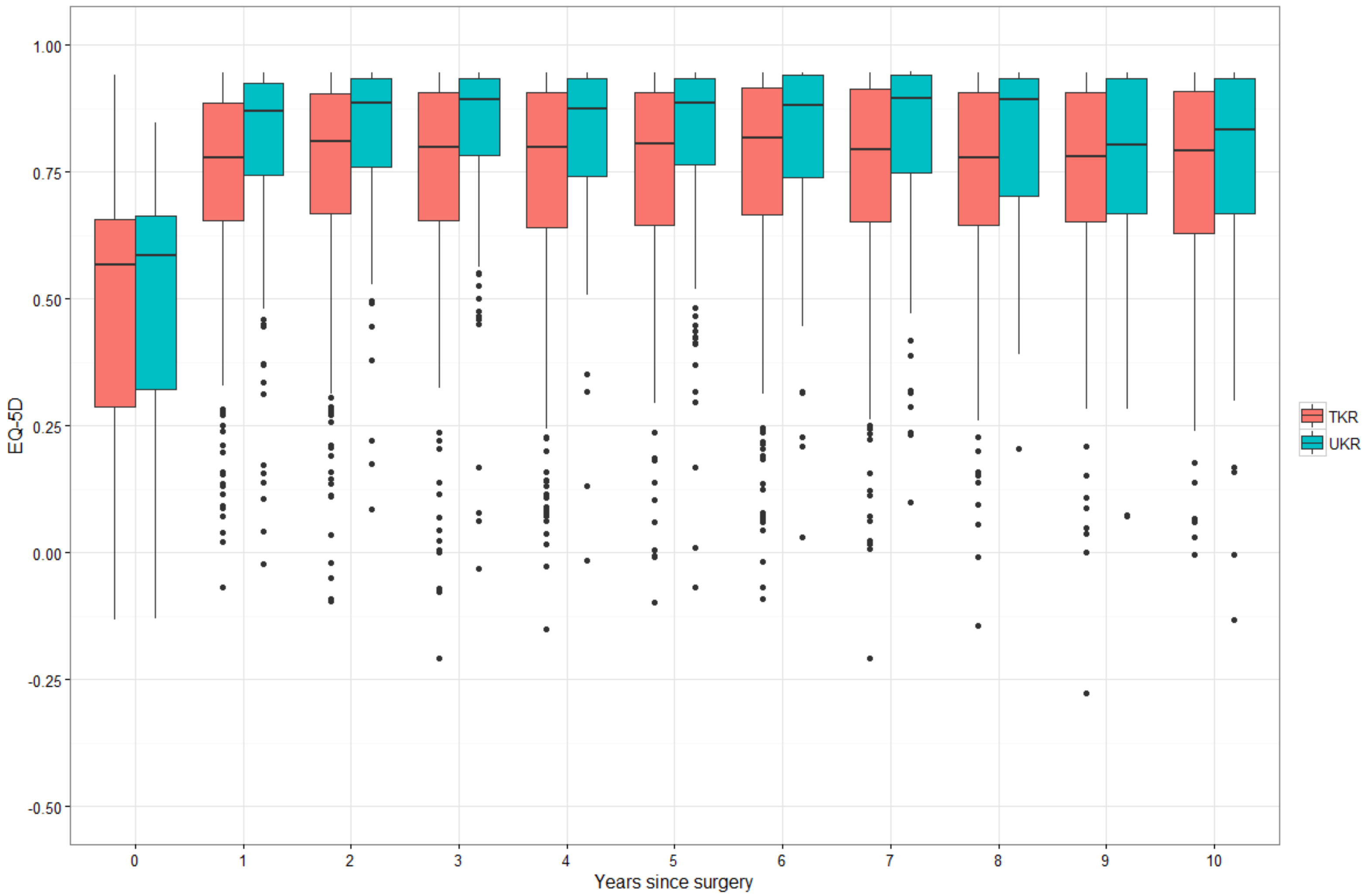

APPENDIX 3

Table A.3.1. Factors associated with Patient-reported outcome Scores over 10 years following surgery

	OKS pain component (0 to 28) *Estimate (95% CI)*	OKS function component (0 to 20) *Estimate (95% CI)*	OKS (0 to 48) *Estimate (95% CI)*	EQ-5D (-0.59 to 1) *Estimate (95% CI)*
Surgery (UKR)	1.74 (1.14 – 2.35) ***	2.23 (1.82 – 2.65) ***	4.04 (3.07 – 5.01) ***	0.073 (0.054 – 0.092) ***
Years since surgery	-0.09 (-0.12 – -0.05) ***	-0.11 (-0.14 – -0.09) ***	-0.19 (-0.25 – -0.14) ***	-0.004 (-0.005 – -0.003) ***
Surgery (UKR)* Years since surgery	0.11 (0.05 – 0.17) ***	0.03 (-0.01 – 0.07)	0.13 (0.04 – 0.21) **	0.002 (-0.000 – 0.003)
Age group				
<60	-1.26 (-2.02 – -0.50) **	-0.27 (-0.79 – 0.25)	-1.45 (-2.68 – -0.21) *	-0.031 (-0.055 – -0.008) **
60 to 70	-	-	-	-
70 to 80	-0.46 (-1.10 – 0.18)	-0.79 (-1.23 – -0.36) ***	-1.38 (-2.41 – -0.34) **	-0.027 (-0.046 – -0.007) **
80+	0.31 (-0.84 – 1.45)	-0.97 (-1.76 – -0.18) *	-0.75 (-2.62 – 1.11)	-0.024 (-0.059 – 0.011)
Gender (male)	-0.30 (-0.86 – 0.25)	0.19 (-0.20 – 0.58)	-0.17 (-1.10 – 0.75)	0.000 (-0.017 – 0.017)
Pre-operative OKS pain component	0.32 (0.26 – 0.37) ***			
Pre-operative OKS function component		0.46 (0.41 – 0.51) ***		
Pre-operative OKS			0.40 (0.35 – 0.46) ***	
Pre-operative EQ-5D				0.265 (0.226 – 0.304) ***
R2	0.762	0.807	0.805	0.773

** p<.05 ** p<.01 *** p<.001. OKS: Oxford knee score; UKR: Unicompartmental knee replacement; TKR: Total knee replacement; CI: confidence intervals*

APPENDIX 4

Table A.4.1. Factors associated with patient-reported outcome scores at 1 year following surgery

	OKS pain component (0 to 28) *Estimate (95% CI)*	OKS function component (0 to 20) *Estimate (95% CI)*	OKS (0 to 48) *Estimate (95% CI)*	EQ-5D (-0.59 to 1) *Estimate (95% CI)*
Surgery (UKR)	1.62 (0.61 – 2.63) **	1.93 (1.26 – 2.61) ***	4.07 (2.58 – 5.57) ***	0.06 (0.03 – 0.09) ***
Age group				
<60	-0.89 (-2.12 – 0.34)	-0.20 (-1.03 – 0.63)	-0.92 (-2.78 – 0.93)	-0.01 (-0.05 – 0.03)
60 to 70	-	-	-	-
70 to 80	0.40 (-0.79 – 1.58)	0.13 (-0.67 – 0.94)	0.49 (-1.36 – 2.34)	0.02 (-0.02 – 0.05)
80+	1.41 (-1.26 – 4.09)	0.80 (-1.02 – 2.62)	0.59 (-3.48 – 4.67)	0.05 (-0.04 – 0.13)
Gender (male)	-0.48 (-1.48 – 0.52)	0.02 (-0.68 – 0.73)	-0.62 (-2.19 – 0.96)	0.01 (-0.03 – 0.04)
Pre-operative OKS pain component	0.35 (0.24 – 0.46) ***			
Pre-operative OKS function component		0.44 (0.34 – 0.55) ***		
Pre-operative OKS			0.38 (0.27 – 0.49) ***	
Pre-operative EQ-5D				0.23 (0.15 – 0.31) ***
R2	0.115	0.226	0.157	0.120

** p<.05 ** p<.01 *** p<.001. OKS: Oxford knee score; UKR: Unicompartmental knee replacement; TKR: Total knee replacement; CI: confidence intervals*

Table A.4.2. Factors associated with patient-reported outcome scores at 10 years following surgery

	OKS pain component (0 to 28) *Estimate (95% CI)*	OKS function component (0 to 20) *Estimate (95% CI)*	OKS (0 to 48) *Estimate (95% CI)*	EQ-5D (-0.59 to 1) *Estimate (95% CI)*
Surgery (UKR)	1.67 (0.57 – 2.78) **	2.09 (1.27 – 2.91) ***	4.54 (2.90 – 6.18) ***	0.07 (0.03 – 0.10) ***
Age group				
<60	-0.65 (-2.00 – 0.69)	0.30 (-0.70 – 1.31)	-0.03 (-2.07 – 2.00)	-0.01 (-0.06 – 0.03)
60 to 70	-	-	-	-
70 to 80	-1.12 (-2.42 – 0.17)	-0.76 (-1.74 – 0.21)	-1.83 (-3.86 – 0.20)	-0.05 (-0.09 – -0.01) *
80+	0.55 (-2.38 – 3.48)	-2.43 (-4.63 – -0.22) *	-3.09 (-7.57 – 1.38)	-0.07 (-0.17 – 0.03)
Gender (male)	-0.09 (-1.18 – 1.01)	0.25 (-0.61 – 1.11)	-0.21 (-1.94 – 1.52)	0.01 (-0.03 – 0.04)
Pre-operative OKS pain component	0.39 (0.27 – 0.51) ***			
Pre-operative OKS function component		0.56 (0.43 – 0.68) ***		
Pre-operative OKS			0.47 (0.35 – 0.59) ***	
Pre-operative EQ-5D				0.29 (0.20 – 0.38) ***
R2	0.105	0.231	0.170	0.135

** p<.05 ** p<.01 *** p<.001. OKS: Oxford knee score; UKR: Unicompartmental knee replacement; TKR: Total knee replacement; CI: confidence intervals*

APPENDIX 5

Table A.5.1. Factors associated with successful patient-reported outcomes over 10 years following surgery

	OKS categories			*Change in OKS*		*Change in EQ-5D*	
	'Excellent' (>41)	'Excellent' or 'good' (>34)	'Excellent', 'good' or 'fair' (>27)	Achieve MCID (≥4)	No change or improvement (≥0)	Achieve MCID (≥0.074)	No change or improvement (≥0)
Surgery (UKR)	4.57 (3.20 – 6.54) ***	3.81 (2.47 – 5.87) ***	3.49 (2.05 – 5.95) ***	2.87 (1.77 – 4.66) ***	3.03 (1.66 – 5.54) ***	2.49 (1.63 – 3.80) ***	2.10 (1.24 – 3.56) **
Years since surgery	1.00 (0.96 – 1.04)	0.91 (0.87 – 0.96) ***	0.91 (0.86 – 0.97) **	0.94 (0.89 – 1.00)	0.99 (0.92 – 1.07)	0.94 (0.89 – 0.99) *	0.91 (0.85 – 0.97) **
Age group							
<60	0.77 (0.42 – 1.40)	0.42 (0.21 – 0.87) *	0.32 (0.14 – 0.77) *	0.28 (0.12 – 0.65) **	0.36 (0.13 – 0.96) *	0.36 (0.17 – 0.77) **	0.21 (0.09 – 0.53) **
60 to 70							
70 to 80	0.93 (0.56 – 1.54)	1.09 (0.58 – 2.04)	1.15 (0.52 – 2.56)	1.17 (0.56 – 2.44)	2.31 (0.91 – 5.88)	1.08 (0.57 – 2.04)	1.21 (0.53 – 2.80)
80+	1.08 (0.43 – 2.70)	1.28 (0.40 – 4.07)	0.87 (0.21 – 3.66)	0.87 (0.25 – 3.06)	1.13 (0.21 – 6.13)	0.59 (0.20 – 1.79)	0.62 (0.15 – 2.63)
Years since surgery**<60*	1.03 (0.96 – 1.10)	1.09 (1.00 – 1.18) *	1.11 (1.01 – 1.23) *	1.16 (1.04 – 1.29) **	1.06 (0.94 – 1.21)	1.14 (1.03 – 1.26) *	1.22 (1.08 – 1.38) **
Years since surgery**60 to 70*							
Years since surgery**70 to 80*	0.91 (0.86 – 0.97) **	0.91 (0.85 – 0.98) *	0.92 (0.84 – 1.02)	0.92 (0.84 – 1.01)	0.83 (0.73 – 0.93) **	0.89 (0.82 – 0.97) **	0.90 (0.81 – 1.00)
Years since surgery**80+*	0.86 (0.76 – 0.98) *	0.91 (0.79 – 1.05)	0.98 (0.82 – 1.18)	0.89 (0.75 – 1.04)	0.94 (0.74 – 1.20)	0.96 (0.83 – 1.13)	0.98 (0.80 – 1.20)
Gender (male)	1.11 (0.77 – 1.61)	0.91 (0.58 – 1.43)	0.84 (0.49 – 1.44)	0.78 (0.47 – 1.28)	0.67 (0.36 – 1.22)	0.96 (0.62 – 1.47)	0.91 (0.54 – 1.55)

Pre-operative OKS	1.13 (1.11 – 1.16) ***	1.14 (1.11 – 1.17) ***	1.15 (1.11 – 1.19) ***	0.94 (0.91 – 0.97) ***	0.93 (0.90 – 0.97) **		
Pre-operative EQ-5D						0.01 (0.00 – 0.04) ***	0.04 (0.01 – 0.16) ***

*** p<.01   *** p<.001. OR: Odds ratio (OR) based on mixed effects logistic regression controlling for years since surgery, age group, gender and pre-operative scores (Appendix 4). Kalairajah OKS categories are based on absolute scores while change in OKS and EQ-5D compare patients' post-operative scores with their pre-operative score. Minimally clinically important difference; MCID: OKS: Oxford knee score; UKR: Unicompartmental knee replacement; CI: confidence intervals.*

APPENDIX 6

Table A.6.1. Patient characteristics before and after matching with no caliper specified

	Before matching			After matching		
	TKR	UKR	Standardised Mean Difference	TKR	UKR	Standardised Mean Difference
N	1,980	675		675	675	
Age (mean, SD)	70.4 (8.17)	66.82 (9.56)	0.4	67.42 (8.41)	66.82 (9.56)	0.07
Gender: male (n, %)	864 (44%)	350 (52%)	0.17	318 (47%)	350 (52%)	0.09
Pre-op OKS pain score (mean, SD)	9.61 (4.62)	12.63 (5.17)	0.62	11.8 (4.86)	12.63 (5.17)	0.17
Pre-op OKS function score (mean, SD)	8.57 (3.51)	11.95 (4.05)	0.89	11.14 (3.47)	11.95 (4.05)	0.21
Pre-op OKS total score (mean, SD)	18.18 (7.5)	24.59 (8.69)	0.79	22.93 (7.71)	24.59 (8.69)	0.2
Pre-op EQ5D baseline (median, IQR)	0.43 (0.16 to 0.6)	0.61 (0.37 to 0.68)	0.62	0.58 (0.34 to 0.66)	0.61 (0.37 to 0.68)	0.13

TKR: total knee replacement, UKR: unicompartmental knee replacement. IQR: interquartile range. Standardised Mean Difference compares the difference in means in units of the pooled standard deviation.

Table A.6.2. Effect of patient and surgical factors on patient-reported outcome measures over 10 years following surgery after matching with no caliper specified

	OKS pain component (0 to 28) *Estimate (95% CI)*	OKS function component (0 to 20) *Estimate (95% CI)*	OKS (0 to 48) *Estimate (95% CI)*	EQ-5D (-0.59 to 1) *Estimate (95% CI)*
Surgery (UKR)	1.40 (0.85 – 1.94) ***	1.94 (1.57 – 2.31) ***	3.34 (2.47 – 4.21) ***	0.06 (0.04 – 0.08) ***
Years since surgery	-0.09 (-0.13 – -0.06) ***	-0.13 (-0.15 – -0.10) ***	-0.22 (-0.27 – -0.17) ***	-0.00 (-0.01 – -0.00) ***
Surgery (UKR)* Years since surgery	0.12 (0.07 – 0.17) ***	0.05 (0.01 – 0.08) **	0.16 (0.09 – 0.24) ***	0.00 (0.00 – 0.00) **
Age group				
<60	-1.05 (-1.72 – -0.39) **	-0.24 (-0.69 – 0.21)	-1.19 (-2.27 – -0.12) *	-0.02 (-0.04 – -0.00) *
60 to 70	-	-	-	-
70 to 80	-0.63 (-1.20 – -0.05) *	-0.80 (-1.18 – -0.41) ***	-1.46 (-2.39 – -0.53) **	-0.03 (-0.05 – -0.01) **
80+	0.35 (-0.75 – 1.44)	-0.87 (-1.61 – -0.14) *	-0.62 (-2.38 – 1.14)	-0.02 (-0.06 – 0.01)
Gender (male)	-0.11 (-0.62 – 0.39)	0.38 (0.04 – 0.73) *	0.23 (-0.60 – 1.06)	0.01 (-0.01 – 0.02)
Pre-operative OKS pain component	0.28 (0.23 – 0.33) ***			
Pre-operative OKS function component		0.41 (0.36 – 0.45) ***		
Pre-operative OKS			0.35 (0.30 – 0.41) ***	
Pre-operative EQ-5D				0.24 (0.21 – 0.28) ***
R2	0.743	0.793	0.785	0.753

** p<.05 ** p<.01 *** p<.001. OKS: Oxford knee score; EQ-5D: EuroQol 5 domain questionnaire; UKR: Unicompartmental knee replacement; TKR: Total knee replacement; CI: confidence intervals*

APPENDIX 7

Table A.7.1. Patient characteristics before and after matching with only KAT knees done by a consultant or associate specialist/ staff grade included in the analysis

	Before matching			After matching		
	TKR	UKR	Standardised Mean Difference	TKR	UKR	Standardised Mean Difference
N	1,428	675		564	564	
Age (mean, SD)	70.25 (8.37)	66.82 (9.56)	0.38	67.76 (8.79)	67.8 (9.13)	0
Gender: male (n, %)	624 (44%)	350 (52%)	0.16	257 (46%)	280 (50%)	0.08
Pre-op OKS pain score (mean, SD)	9.61 (4.62)	12.63 (5.17)	0.62	11.28 (5.05)	11.72 (4.81)	0.09
Pre-op OKS function score (mean, SD)	8.59 (3.48)	11.95 (4.05)	0.89	10.61 (3.53)	10.98 (3.61)	0.1
Pre-op OKS total score (mean, SD)	18.19 (7.48)	24.59 (8.69)	0.79	21.89 (7.97)	22.72 (7.9)	0.1
Pre-op EQ5D baseline (median, IQR)	0.42 (0.17 to 0.6)	0.61 (0.37 to 0.68)	0.62	0.56 (0.28 to 0.65)	0.58 (0.31 to 0.66)	0.08

TKR: total knee replacement, UKR: unicompartmental knee replacement. IQR: interquartile range. Standardised Mean Difference compares the difference in means in units of the pooled standard deviation.

Table A.7.2. Effect of patient and surgical factors on patient-reported outcome measures over 10 years following surgery after matching with only KAT knees done by a consultant or associate specialist/ staff grade included in the analysis

	OKS pain component (0 to 28) *Estimate (95% CI)*	OKS function component (0 to 20) *Estimate (95% CI)*	OKS (0 to 48) *Estimate (95% CI)*	EQ-5D (-0.59 to 1) *Estimate (95% CI)*
Surgery (UKR)	1.75 (1.13 – 2.37) ***	2.16 (1.75 – 2.58) ***	3.87 (2.89 – 4.85) ***	0.07 (0.05 – 0.09) ***
Years since surgery	-0.06 (-0.10 – -0.03) **	-0.12 (-0.14 – -0.09) ***	-0.18 (-0.24 – -0.13) ***	-0.00 (-0.01 – -0.00) ***
Surgery (UKR)* Years since surgery	0.08 (0.02 – 0.14) *	0.03 (-0.01 – 0.07)	0.11 (0.02 – 0.19) *	0.00 (-0.00 – 0.00)
Age group				
<60	-1.24 (-2.02 – -0.46) **	-0.30 (-0.82 – 0.23)	-1.45 (-2.71 – -0.19) *	-0.03 (-0.06 – -0.01) **
60 to 70	-	-	-	-
70 to 80	-0.73 (-1.38 – -0.08) *	-0.99 (-1.43 – -0.56) ***	-1.80 (-2.84 – -0.75) **	-0.04 (-0.06 – -0.02) **
80+	-0.87 (-1.97 – 0.22)	-1.45 (-2.18 – -0.71) ***	-2.46 (-4.22 – -0.69) **	-0.06 (-0.10 – -0.03) ***
Gender (male)	-0.57 (-1.15 – 0.00)	0.08 (-0.31 – 0.48)	-0.59 (-1.53 – 0.35)	-0.00 (-0.02 – 0.01)
Pre-operative OKS pain component	0.32 (0.26 – 0.38) ***			
Pre-operative OKS function component		0.47 (0.41 – 0.52) ***		
Pre-operative OKS			0.41 (0.35 – 0.47) ***	
Pre-operative EQ-5D				0.26 (0.22 – 0.30) ***
R2	0.751	0.803	0.795	0.774

** p<.05 ** p<.01 *** p<.001. OKS: Oxford knee score; EQ-5D: EuroQol 5 domain questionnaire; UKR: Unicompartmental knee replacement; TKR: Total knee replacement; CI: confidence intervals*
